# c-FLIP Protects Eosinophils from TNF-α-Mediated Cell Death *In Vivo*

**DOI:** 10.1371/journal.pone.0107724

**Published:** 2014-10-21

**Authors:** Claire Gordy, Jie Liang, Heather Pua, You-Wen He

**Affiliations:** Department of Immunology, Duke University Medical Center, Durham, North Carolina, United States of America; Toho University School of Medicine, Japan

## Abstract

Understanding the signals that regulate eosinophil survival and death is critical to developing new treatments for asthma, atopy, and gastrointestinal disease. Previous studies suggest that TNF-α stimulation protects eosinophils from apoptosis, and this TNF-α-mediated protection is mediated by the upregulation of an unknown protein by NF-κB. Here, we show for the first time that eosinophils express the caspase 8-inhibitory protein c-FLIP, and c-FLIP expression is upregulated upon TNF-α stimulation. Considering that c-FLIP expression is regulated by NF-κB, we hypothesized that c-FLIP might serve as the “molecular switch” that converts TNFRI activation to a pro-survival signal in eosinophils. Indeed, we found that one c-FLIP isoform, c-FLIP_L_, is required for mouse eosinophil survival in the presence of TNF-α both *in vitro* and *in vivo*. Importantly, our results suggest c-FLIP as a potential therapeutic target for the treatment of eosinophil-mediated disease.

## Introduction

Although eosinophils were one of the first types of leukocytes to be described, their physiologic and pathologic functions remain poorly understood. After development from hematopoietic progenitors, mature eosinophils circulate for less than a day before homing to the gastrointestinal tract, thymus, mammary glands, or uterus, where they are thought to function in antigen presentation, T cell polarization, thymocyte selection, mammary gland development, and reproduction [1]. Inflammation triggers increased production of eosinophils in the bone marrow and recruitment of these eosinophils to the site of inflammation, where they regulate T cell responses and directly interact with pathogens through the release of inflammatory granule proteins [1].

For many years, studies have suggested that eosinophils function in the clearance of helminth parasites; however, recent studies suggest that they may instead promote the survival of certain helminthes [2], [3]. Accumulating evidence points to a role for eosinophils in the clearance of bacterial and viral pathogens; however, these functions can result in pathology in the lung [4].

In addition to these proposed functions in promoting immunity and maintaining homeostasis, eosinophils have been implicated in numerous pathological states, including allergy, asthma, and gastrointestinal disease. The mechanisms by which eosinophils mediate these diseases remain unclear; however, developing strategies to deplete or inhibit eosinophils is a subject of intense research.

Most current anti-eosinophil therapies function by decreasing eosinophil numbers at steady state. This is achieved by inhibiting eosinophil development (interferon-α, anti-IL-5 monoclonal antibody therapy) or eosinophil survival (corticosteroids, anti-IL-5 monoclonal antibody therapy) [5]. Corticosteroids, which remain the most effective and most commonly used therapy in eosinophil-mediated diseases, act on many types of cells and thus have toxic effects [5]. Therapies that target IL-5 or IL-5R, such as mepolizumab, reslizumab, and benralizumab, are specific, but their efficacy appears to vary widely in different subgroups of patients [5]. Moreover, despite the decreases in circulating eosinophils that have been observed in clinical trials of these drugs, many patients display residual tissue eosinophilia [5].

Importantly, because these drugs act on both inflammatory eosinophils that have accumulated in peripheral tissues and eosinophils involved in homeostatic processes in the gut, thymus, and reproductive organs, the effects of these therapies on the beneficial functions of eosinophils must be considered.

Another class of eosinophil-targeted therapies functions by inhibiting eosinophil recruitment to peripheral tissues. These drugs, which target adhesion molecules, chemokines, and their receptors, have shown limited efficacy in clinical trials, and require phenotypic and genotypic screening to identify patients who are likely to respond [5].

Because the current therapies either work only in a subset of patients or deplete eosinophils not involved in inflammatory processes, a new approach is required. To effectively target pathogenic eosinophils without depleting eosinophils performing beneficial functions requires a better understanding of signals that regulate eosinophil survival under homeostatic and inflammatory conditions.

TNF-α, a pleiotropic cytokine that can mediate cell death or cell survival and proliferation depending on cell type and environment, promotes the survival of eosinophils through an NF-κB-dependent mechanism [6], [7]. Inhibition of NF-κB in eosinophils converts TNF-α to a pro-apoptotic signal that results in caspase-dependent cell death; however, the downstream mediators of NF-κB-dependent eosinophil survival have not been identified [6], [7].

c-FLIP, an inhibitor of caspase 8 activity, is upregulated by NF-κB signaling in human lung fibroblast, fibrosarcoma, cervical cancer, skeletal muscle, and B lymphoma cells [8], [9], [10], [11]. We previously showed that c-FLIP is required for the survival of macrophages but not of neutrophils; however, the function of c-FLIP in eosinophils has not been studied [12]. Based on its function and regulation in other cell types, we hypothesized that c-FLIP might function as the molecular switch that converts TNF-α from a pro-apoptotic signal to a pro-survival signal in eosinophils.

Indeed, we report for the first time that eosinophils express c-FLIP, and c-FLIP expression is upregulated upon TNF-α stimulation. Although the deletion of c-FLIP has no effect on eosinophil survival in the absence of inflammatory signals, c-FLIP is required for the survival of TNF-α-stimulated eosinophils *in vitro* and *in vivo*. Together, our data reveal a differential requirement for c-FLIP in unstimulated and inflamed eosinophils. Future research in human eosinophils will reveal whether c-FLIP is a relevant target for anti-eosinophil therapies.

## Results

### TNF-α upregulates c-FLIP in eosinophils

Previous studies showed that inhibiting NF-κB activity or protein synthesis converted TNF-α from a pro-survival signal to a pro-apoptosis signal in eosinophils [6], [7]. Because NF-κB activation upregulates the expression of the anti-apoptotic protein c-FLIP in many cell types [8], [9], [10], [11], we hypothesized that TNF-α stimulation may upregulate c-FLIP in eosinophils.

**Figure 1:**
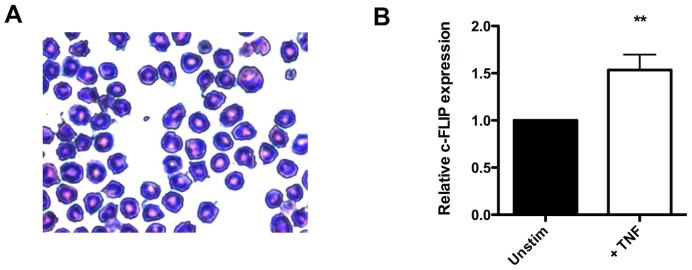
TNF-α upregulates c-FLIP in BMDE. A: Representative cytospin of BMDE. B: c-FLIP expression was measured by qPCR in WT BMDE before and after stimulation with 50 ng/ml TNF-α for 24 h. The data were obtained in four independent experiments. **, p<0.01 (Student's *t*-test).

Due to the paucity of circulating eosinophils *in vivo*, we examined the mRNA expression of c-FLIP in bone marrow-derived eosinophils (BMDEs) from wild type mice by qPCR (Fig. 1A, B). We detected the expression of c-FLIP mRNA in unstimulated BMDEs, and treatment with TNF-α resulted in a 50% increase in c-FLIP expression (Fig. 1B). Importantly, these data provide the first evidence that eosinophils express c-FLIP [13]. Moreover, the upregulation of c-FLIP in TNF-α-stimulated cells suggests that c-FLIP may function as the molecular switch that determines the fate of eosinophils following TNF-α stimulation.

### c-FLIP_L_ protects eosinophils from TNF-α-mediated death *in vitro*

**Figure 2:**
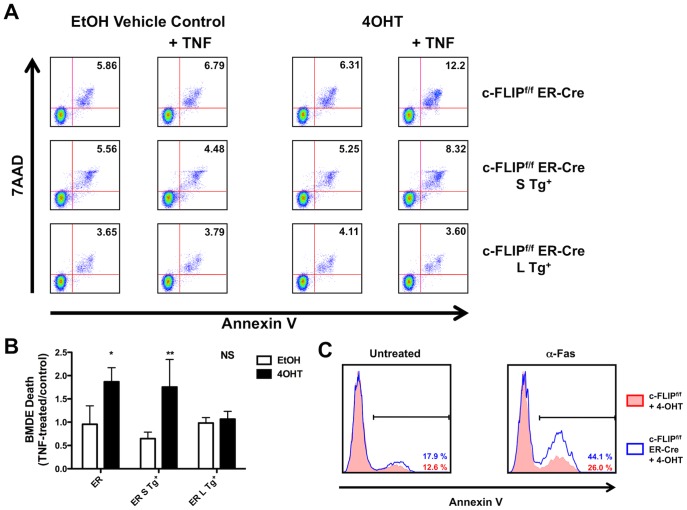
c-FLIP_L_ protects BMDE from TNF-α-induced apoptosis. A-B: BMDE from c-FLIP^f/f^ Lysm-Cre, c-FLIP^f/f^ Lysm-Cre S Tg^+^, or c-FLIP^f/f^ Lysm-Cre L Tg^+^ mice were cultured with 4-OHT or EtOH for 4 days and then stimulated or not with 50 ng/ml TNF-α for 24 h. Apoptosis rates were determined by measuring the percent of Annexin V^+^/7AAD^+^ cells within the CD11b^int^ CCR3^+^ population by flow cytometry. Representative FACS plots are shown in A. Numbers indicate the frequency of Annexin V^+^/7AAD^+^ eosinophils in each sample. B: The effect of TNF-α on apoptosis was determined by dividing the percent Annexin V^+^/7AAD^+^ cells in each TNF-α-treated sample by that in the paired control sample. The data were obtained in three independent experiments. Error bars represent standard deviations. *, p<0.05; **, p<0.01 (Student's *t*-test). C: Frequency of Annexin V^+^ BMDE with (right) or without (left) treatment with anti-Fas antibody. Filled red histograms represent 4-OHT-treated c-FLIP^f/f^ cells, and open blue histograms represent 4-OHT-treated c-FLIP^f/f^ ER-Cre cells.

To address the role of c-FLIP in eosinophil survival, we generated BMDEs from c-FLIP^f/f^ ER-Cre bone marrow, allowing us to delete c-FLIP in a highly pure eosinophil population *in vitro* (Figure 1A). Because these cells express the tamoxifen-inducible ER-Cre recombinase, treatment with 4-hydroxy-tamoxifen (4-OHT) results in deletion of the loxP-flanked c-FLIP alleles (Table 1). In the absence of stimulation, 4-OHT-treated eosinophils displayed no survival defect compared to vehicle-treated controls; however, TNF-α stimulation resulted in a ∼2-fold increase in apoptosis in c-FLIP-deleted BMDEs (Fig. 2A, B).

**Table 1:** Summary of mouse genetic models used for *in vitro* experiments.

Genotype	Endogenous c-FLIP status	Cre expression	BAC Transgenes	Before 4-OHT treatment	After 4-OHT
**c-FLIP^f/f^**	Both endogenous c-FLIP alleles flanked by LoxP sites.	No Cre recombinase.	None.	Wild type.	Wild type.
**c-FLIP^f/f^ ER-Cre**		Tamoxifen (4OHT)-inducible Cre recombinase expressed in all cells			Completely lack c-FLIP.
**c-FLIP^f/f^ ER-Cre c-FLIP_S_ BAC Tg^+^**			c-FLIP_S_ isoform-specific BAC transgene expressed in all cells.		Express only c-FLIP_S_.
**c-FLIP^f/f^ ER-Cre c-FLIP_L_ BAC Tg^+^**			c-FLIP_L_ isoform-specific BAC transgene expressed in all cells.		Express only c-FLIP_L_.

To compare the roles of the two c-FLIP isoforms in protecting eosinophils from TNF-α-mediated cell death, we performed the same experiment in BMDEs generated from c-FLIP^f/f^ ER-Cre mice expressing an isoform-specific bacterial artificial chromosome (BAC) transgene (c-FLIP^f/f^ ER-Cre S Tg^+^, which results in c-FLIP_L_-deficient cells, or c-FLIP^f/f^ ER-Cre L Tg^+^, which results in c-FLIP_S_-deficient cells) (Table 2). Interestingly, c-FLIP_L_-deficient eosinophils displayed increased apoptosis upon TNF-α stimulation, but c-FLIP_S_-deficient eosinophils died at similar rates to unstimulated controls (Fig. 2A, B). These data demonstrate that the c-FLIP_L_ isoform is required to prevent TNF-α-mediated eosinophil death *in vitro*.

**Table 2:** Summary of mouse genetic models used for *in vivo* experiments.

Genotype	Endogenous c-FLIP status	Cre expression	BAC Transgenes	Other gene deletions	Myeloid cells	Non-myeloid cells
**c-FLIP^f/f^**	Both endogenous c-FLIP alleles flanked by LoxP sites.	No Cre recombinase.	None.	None.	Wild type.	Wild type.
**c-FLIP^f/f^ Lysm-Cre**		Cre recombinase expressed in myeloid cells; endogenous c-FLIP deleted in myeloid cells *in vivo*.			Completely lack c-FLIP.	Wild type.
**c-FLIP^f/f^ Lysm-Cre c-FLIP_S_ BAC Tg^+^**			c-FLIP_S_ isoform-specific BAC transgene expressed in all cells.		Express only c-FLIP_S_.	Wild type.
**c-FLIP^f/f^ Lysm-Cre c-FLIP_L_ BAC Tg^+^**			c-FLIP_L_ isoform-specific BAC transgene expressed in all cells.		Express only c-FLIP_L_.	Wild type.
**c-FLIP^f/f^ Lysm-Cre TNF-α^−/−^**			None.	TNF-α knocked out in all cells.	Completely lack c-FLIP and TNF-α.	Completely lack TNF-α. Wild type c-FLIP expression.

### c-FLIP is required for eosinophil survival during inflammation *in vivo*

We next examined the role of c-FLIP in promoting eosinophil survival *in vivo* using the c-FLIP^f/f^ Lysm-Cre mouse model, which lacks the expression of both c-FLIP_L_ and c-FLIP_S_ in myeloid cells. c-FLIP^f/f^ Lysm-Cre mice display a complex phenotype characterized by a loss of multiple macrophage populations and secondary neutrophilia [12]; however, the eosinophil population has not previously been studied in these mice.

**Figure 3:**
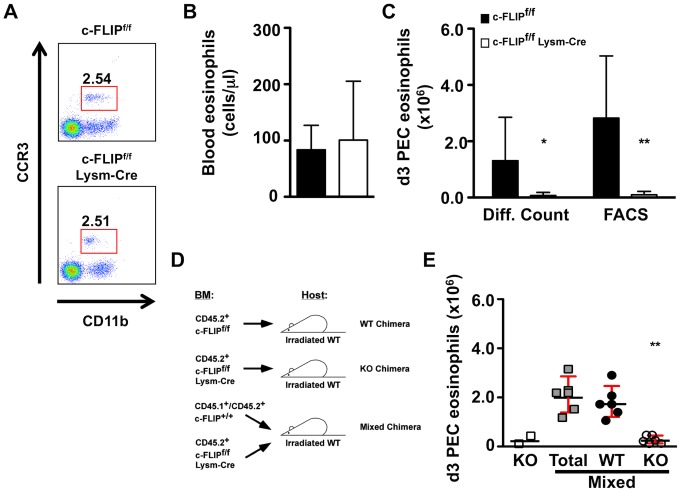
c-FLIP is required for the survival of thioglycollate-elicited eosinophils *in vivo*. A–B: Frequency of CD11b^int^ CCR3^+^ eosinophils in peripheral blood from c-FLIP^f/f^ and c-FLIP^f/f^ Lysm-Cre mice. Representative FACS plots are shown in A. The absolute number of eosinophils/µl peripheral blood is plotted in B. Bars represent geometric means, and error bars represent 95% CI. NS, not significant (Mann-Whitney). C: Absolute numbers of eosinophils in day 3 thioglycollate-elicited PEC samples as determined by differential counting or flow cytometry (CD11b^int^ CCR3^+^). Bars represent geometric means, and error bars represent 95% CI. n = 10 (c-FLIP^f/f^), n = 8 (c-FLIP^f/f^ Lysm-Cre). *, p<0.05; **, p<0.01 (Mann-Whitney). D: Experimental setup for bone marrow chimera experiments. E: Absolute numbers of eosinophils in day 3 thioglycollate-elicited PEC samples in bone marrow chimeric mice as determined by differential counting. Each data point represents an individual mouse (n = 2 KO chimeras, n = 6 mixed chimeras). For mixed chimeras, gray squares represent the total number of PEC eosinophils, black circles represent the number of PEC eosinophils from the c-FLIP^f/f^ donor, and open circles represent the number of PEC eosinophils from the c-FLIP^f/f^ Lysm-Cre donor. Horizontal lines represent geometric means, and error bars represent 95% CI. **, p<0.01 vs. total PEC eosinophils and vs. WT PEC eosinophils in mixed chimeric mice (Mann-Whitney).

Under steady-state conditions, few eosinophils can be detected in the blood of wild type mice, as the vast majority of these cells reside within the tissues. Indeed, in wild type (c-FLIP^f/f^) mice, we detected a small but distinct eosinophil population, defined as CD11b^int^ Gr1^int^ CCR3^+^ cells, in the peripheral blood (Fig. 3A, B). Similar to our findings in unstimulated BMDEs, the loss of c-FLIP did not affect the number of circulating eosinophils *in vivo* (Fig. 3A, B).

To study the function of c-FLIP in eosinophils during inflammation, we employed the thioglycollate-elicited peritonitis model. At steady state, the peritoneum contains a small number of leukocytes, most of which are macrophages and B cells. Following intraperitoneal injection of thioglycollate, peritoneal inflammation results in an early influx of neutrophils that is followed by the death of the resident large peritoneal macrophage population, an increase in small peritoneal macrophages, clearance of the infiltrated neutrophils, and an influx of eosinophils by day 3 post-injection [14]. We thus assessed the absolute number of peritoneal eosinophils in c-FLIP^f/f^ and c-FLIP^f/f^ Lysm-Cre mice by flow cytometry on day 3 post-injection. In contrast to our observations in the blood of untreated mice, we observed a dramatic decrease in the number of thioglycollate-elicited peritoneal eosinophils in c-FLIP^f/f^ Lysm-Cre mice (Fig. 3C). Importantly, differential counting based on cell morphology produced similar results, validating the use of CD11b, Gr1, and CCR3 as eosinophil markers *in vivo* (Fig. 3C). These findings suggest that unlike IL-5, which is dispensable for eosinophil survival outside the bone marrow [15], c-FLIP expression is required for eosinophils to survive in peripheral tissues.

We previously reported that the majority of the defects observed in c-FLIP^f/f^ Lysm-Cre mice, including neutrophilia, decreased body weight, and splenomegaly, were secondary to the loss of macrophages observed in these mice [12]. We therefore considered the possibility that the loss of eosinophils in c-FLIP^f/f^ Lysm-Cre mice was also a secondary effect of the loss of macrophages. To address this question, we used a mixed bone marrow chimera system in which lethally irradiated wild type mice received either c-FLIP^f/f^ Lysm-Cre bone marrow or a 1∶1 mixture of congenically marked c-FLIP^f/f^ Lysm-Cre and wild type bone marrow (Fig. 3D). Twelve weeks after the adoptive transfer, the mice were injected i.p. with thioglycollate, and the presence of eosinophils in the peritoneum was assessed on day 3 post-injection.

Similar to our observations in c-FLIP^f/f^ Lysm-Cre mice, the peritoneal cavities of mice that received only c-FLIP^f/f^ Lysm-Cre bone marrow contained few or no eosinophils (Fig. 3E). In contrast, normal numbers of eosinophils were present in the peritoneal cavities of mice that received a 1∶1 mixture of c-FLIP^f/f^ Lysm-Cre and wild type bone marrow, and nearly 100% of the eosinophils in these mice were derived from wild type donor bone marrow (Fig. 3E). These data indicate that the absence of thioglycollate-elicited peritoneal eosinophils in c-FLIP^f/f^ Lysm-Cre mice is due to the lack of c-FLIP expression in eosinophils, as virtually none of the eosinophils in mixed bone marrow chimeric mice, which are phenotypically normal and display no defects in macrophages or neutrophils, were derived from c-FLIP^f/f^ Lysm-Cre bone marrow.

### c-FLIP-deficient eosinophils die through a TNF-α-mediated pathway *in vivo*

**Figure 4:**
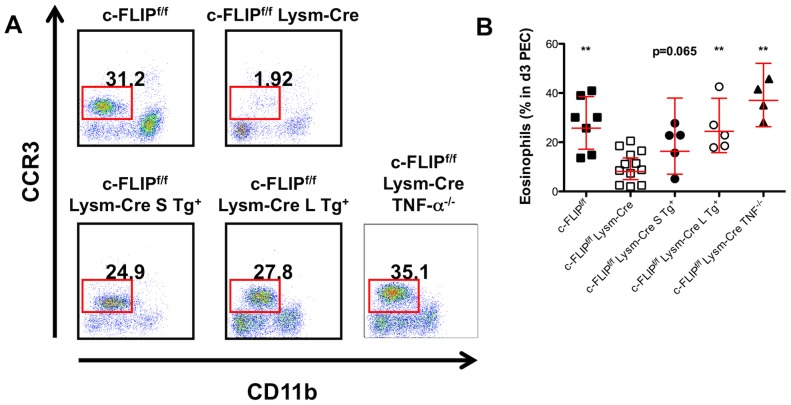
c-FLIP_L_ is required to protect eosinophils from TNF-α-mediated apoptosis *in vivo*. A–B: Frequency of eosinophils in day 3 thioglycollate-elicited PEC from c-FLIP^f/f^, c-FLIP^f/f^ Lysm-Cre, c-FLIP^f/f^ Lysm-Cre S Tg^+^, c-FLIP^f/f^ Lysm-Cre L Tg^+^, c-FLIP^f/f^ Lysm-Cre TNF-α^−/−^ mice. Representative FACS plots are shown in A. Numbers indicate the frequency of CD11b^int^ CCR3^+^ eosinophils in each sample. Each data point in B represents an individual mouse (n = 7 for c-FLIP^f/f^, n = 12 for c-FLIP^f/f^ Lysm-Cre, n = 5 for c-FLIP^f/f^ Lysm-Cre S Tg^+^, n = 5 for c-FLIP^f/f^ Lysm-Cre L Tg^+^, and n = 4 for c-FLIP^f/f^ Lysm-Cre TNF-α^−/−^). Horizontal lines indicate geometric means, and error bars represent 95% CI. **, p<0.01 vs. c-FLIP^f/f^ Lysm-Cre (Mann-Whitney).

Together, these results demonstrate a novel role for c-FLIP in eosinophil survival both *in vitro* and *in vivo*. Moreover, our *in vitro* experiments suggested that c-FLIP may serve as the previously hypothesized “molecular switch” that defines a pro-apoptotic or anti-apoptotic fate downstream of TNFRI signaling in eosinophils. To determine whether the loss of eosinophils observed in c-FLIP^f/f^ Lysm-Cre mice occurred through TNF-α-dependent cell death, we generated c-FLIP^f/f^ Lysm-Cre TNF-α-deficient mice. In support of our hypothesis, c-FLIP^f/f^ Lysm-Cre TNF-α^−/−^ mice had normal numbers of thioglycollate-elicited peritoneal eosinophils, demonstrating that c-FLIP protects eosinophils from TNF-α-mediated death *in vivo* (Fig. 4A, B).

We next aimed to determine whether both c-FLIP isoforms were required for eosinophil survival. To address this question, we generated mice that expressed only c-FLIP_L_ or c-FLIP_S_ in eosinophils by breeding c-FLIP^f/f^ Lysm-Cre mice with mice expressing a c-FLIP_L_ or c-FLIP_S_ isoform-specific bacterial artificial chromosome (BAC) transgene [16]. Expression of either isoform increased the frequency of thioglycollate-elicited peritoneal eosinophils; however, expression of c-FLIP_L_ provided a more complete rescue than did expression of c-FLIP_S_ (Fig. 4A, B). Together with our finding that c-FLIP_L_ conferred complete protection from TNF-α-mediated apoptosis *in vitro*, these results suggest that c-FLIP_L_ serves as the molecular switch that converts TNF-α from a pro-apoptotic signal to a pro-survival signal in eosinophils.

## Discussion

**Figure 5:**
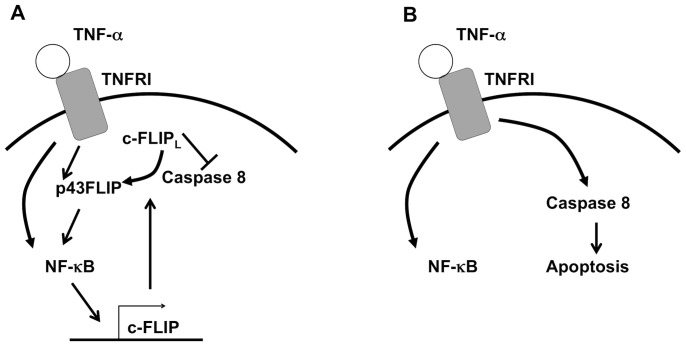
A model for c-FLIP as a molecular switch between pro- and anti-apoptotic TNF-α-mediated signaling in eosinophils. A: Upon TNFRI signaling, c-FLIP_L_ inhibits caspase 8 cleavage. The c-FLIP_L_ cleavage product p43FLIP mediates TNFRI-mediated activation of NF-κB, which upregulates c-FLIP, creating a positive feedback loop that protects eosinophils from TNF-α-mediated apoptosis. B: In the absence of c-FLIP, TNFRI signaling results in caspase 8 cleavage and apoptosis. TNFRI-mediated activation of NF-κB fails to protect eosinophils from apoptosis because NF-κB cannot upregulate c-FLIP.

Together, our results demonstrate a novel role for c-FLIP in protecting eosinophils from apoptosis. TNF-α has previously been reported to mediate anti-apoptotic cell fates in eosinophils in the presence of NF-κB signaling and pro-apoptotic cell fates in its absence [6], [7]; however, the pro-survival mediators downstream of NF-κB have not yet been identified. When considered in the context of these previous reports, our data suggest that upon signaling through TNFRI, NF-κB activation serves to transcriptionally upregulate c-FLIP, thereby protecting eosinophils from extrinsic apoptosis. When this pathway is blocked by inhibiting NF-κB activation or inhibiting protein synthesis, TNF-α-stimulated eosinophils can not upregulate c-FLIP and undergo apoptosis (Fig. 5).

This model is supported by our finding that c-FLIP_L_ promotes eosinophil survival more efficiently than does c-FLIP_S_, as p43FLIP, a caspase 8 cleavage product of c-FLIP_L_, can itself activate the NF-κB signaling pathway [17], [18], [19], thereby initiating a positive feedback loop in which c-FLIP_L_ drives its own upregulation (Fig. 5).

Interestingly, c-FLIP-deficient eosinophils displayed enhanced apoptosis upon stimulation with other extrinsic apoptotic triggers, such as FasL (Fig. 2C). Unlike TNF-α, FasL does not mediate anti-apoptotic effects; accordingly, we found that Fas stimulation increased cell death in wild type cells, and Fas-mediated cell death was increased in c-FLIP-deficient cells. Together, these findings raise the possibility that TNFRI-mediated upregulation of c-FLIP may protect eosinophils from a variety of apoptotic stimuli and may thereby serve as the key regulator of eosinophil survival during inflammation. Future studies of the ability of TNF-α to tune the sensitivity of eosinophils to different types of apoptotic stimuli will shed further light on this observation.

Previous studies of the role of extrinsic apoptosis in spontaneous and glucocorticoid-induced eosinophil apoptosis have produced conflicting results. Whether caspase 8 is activated during spontaneous apoptosis remains unclear, and although caspase 8 is required for Fas-mediated eosinophil apoptosis, inhibiting caspase 8 does not prevent spontaneous or glucocorticoid-mediated apoptosis [20], [21], [22], [23], [24], [25]. Similarly, we found that c-FLIP was not required for the survival of BMDE in the absence of stimulation, and unperturbed c-FLIP^f/f^ Lysm-Cre mice displayed normal numbers of circulating eosinophils. Thus, the role of c-FLIP in protecting eosinophils from apoptosis appears to be restricted to stimulation-induced death.

Importantly, the finding that c-FLIP regulates eosinophil survival under inflammatory conditions presents a new potential therapeutic target in eosinophilic diseases. The therapies currently being pursued fall into two categories – drugs that impede eosinophil production or survival, and drugs that prevent recruitment of eosinophils to peripheral tissues. Thus far, few of these therapies have consistently achieved statistically significant results in clinical trials.

Systemic administration of glucocorticoids thus remains the most common treatment for many eosinophilic diseases. While their mechanisms of action are not fully understood, glucocorticoids induce apoptosis of lymphocytes and eosinophils but not of neutrophils [26], [27], [28]. Although glucocorticoid treatment is effective in many eosinophil-mediated diseases, the wide ranging effects of glucocorticoids raise the possibility of deleterious side effects; thus, understanding the mechanisms by which glucocorticoids induce eosinophil death but enhance neutrophil survival will allow for the development of eosinophil-specific targeted therapies.

Interestingly, glucocorticoids inhibit NF-κB activity in many cell types and reverse TNF-α-mediated eosinophil survival [28], [29], [30], [31], [32], [33], [34], [35], [36]. Our finding that c-FLIP_L_ is required for TNF-α-mediated eosinophil survival suggests that glucocorticoids may exert their pro-apoptotic effect on eosinophils by downregulating c-FLIP. Future studies of the effects of glucocorticoids on c-FLIP expression in eosinophils will shed light on the molecular mechanisms underlying glucocorticoid-mediated eosinophil death and could lead to the development of new targeted therapeutics.

Together, our findings reveal a novel pro-survival pathway in eosinophils. Targeting c-FLIP could result in a new class of anti-eosinophil therapies that cause the death of eosinophils upon recruitment to inflamed tissues. Future studies in human eosinophils will examine the potential therapeutic implications of these findings in conditions including asthma, atopy, and gastrointestinal disease.

## Materials and Methods

### Genetic models

The genetic models used in this study are described in Tables 1 and 2. c-FLIP^f/f^ Lysm-Cre mice and c-FLIP^f/f^ ER-Cre mice were generated as previously described [12]. c-FLIP^f/f^ Lysm-Cre mice were bred with mice expressing bacterial artificial chromosome (BAC) transgenes for c-FLIP_S_ or c-FLIP_L_ [16] to generate c-FLIP^f/f^ Lysm-Cre S Tg^+^ or c-FLIP^f/f^ Lysm-Cre L Tg^+^ mice, respectively. All mice were used at 6–8 weeks of age, except as indicated in the text.

### Isolation of primary cells

Blood samples were collected by tail bleed or by submandibular bleed into tubes containing 10 µl of 1000 U/ml heparin sodium salt in PBS.

Bone marrow was harvested by flushing femurs and tibiae with RPMI using a 10-ml syringe and 27-gauge needle. Bone marrow cells were centrifuged, and red blood cells were lysed with ACK buffer. After red blood cell lysis, the cells were resuspended in 5 ml RPMI containing 5% FBS, filtered through 90-µm nylon mesh, and counted.

Peritoneal eosinophils were elicited by injecting mice i.p. with 1 ml 3% thioglycollate. The mice were sacrificed 3 days post-injection, and PEC were harvested by peritoneal lavage with 10 ml of PBS or RPMI containing 5% FBS using a 10-ml syringe and 21-gauge needle. After red blood cell lysis, the cells were resuspended in 5 ml RPMI containing 5% FBS, filtered through 90-µm nylon mesh, and counted.

### Generation of bone marrow-derived eosinophils

To generate bone marrow-derived eosinophils (BMDE), bone marrow cells were isolated from c-FLIP^f/f^ or c-FLIP^f/f^ ER-Cre mice on day 0. After red blood cell lysis, the cells were resuspended in 10 ml RPMI containing 10% FBS and filtered through 90-µm nylon mesh. The filtered cells were counted and resuspended at a concentration of 10^6^ cells/ml in BMDE medium (RPMI containing 20% FBS, 2 mM L-glutamine, 25 mM HEPES, 1x non-essential amino acids, 1 mM sodium pyruvate, and 50 µM 2-ME) containing 100 ng/ml SCF and 100 ng/ml FLT3L (Peprotech, Rocky Hill, NJ).

The cells were cultured at 37°C in the presence of 5% CO_2_. On day 4, the cells were removed from the flasks by pipetting, centrifuged, and resuspended in an equivalent volume of fresh BMDE medium containing 10 ng/ml IL-5 (Peprotech). The cells were then transferred to new flasks and cultured at 37°C in the presence of 5% CO_2_. On day 8, the cells were again removed from the flasks by pipetting, centrifuged, and resuspended in an equivalent volume of fresh BMDE medium containing 10 ng/ml IL-5 (Peprotech). The cells were then transferred to new flasks and cultured at 37°C in the presence of 5% CO_2_. On days 10, 12, 14, and 16, the cells were removed from the flasks by pipetting, centrifuged, counted, adjusted to a concentration of 10^6^ cells/ml in fresh BMDE medium containing 10 ng/ml IL-5, and cultured at 37°C in the presence of 5% CO_2_. On day 18, mature eosinophils were removed from the flasks by pipetting, centrifuged, and counted.

The purity of eosinophils was assessed by cytospin. The cells were suspended at a concentration of 10^7^/ml in PBS, and 100-µl samples of suspended cells were subjected to cytospin. The slides were allowed to dry and stained using a Hema 3 Stat Pack (Fisher Scientific, Kalamazoo, MI). Images were obtained using a Zeiss Axiovert 200 microscope.

### Quantitative real-time PCR

1×10^6^ wild type BMDE were either stimulated with 50 ng/ml TNF-α (Peprotech, Inc., Rocky Hill, NJ) or left unstimulated in culture for the indicated time. 1×10^5^ cells were used for RNA extraction using RNAqueous-Micro Total RNA Isolation Kit (Invitrogen, AM1931). cDNA was generated using SuperscriptΙΙΙ Reverse Transcriptase (Invitrogen) with Oligo-dT_12-18_ as primers. Real-time PCR was performed using Taqman mRNA assay (probe ID: Mm01255578 for cFlar/cFlip; Mm00607939 for β-actin).

### *In vitro* deletion of c-FLIP and stimulation of BMDE

c-FLIP^f/f^ or c-FLIP^f/f^ ER-Cre BMDE were treated with 200 nM 4-hydroxytamoxifen (4-OHT, Sigma-Aldrich, St. Louis, MO) or an equal volume of ethanol vehicle. Four days after c-FLIP deletion, the cells were stimulated with 50 ng/ml TNF-α for 24 h or with 5 µg/ml anti-Fas antibody for 24 h.

### Flow cytometry

The following antibodies were used to stain cell surface antigens: CCR3, CD11b, B220, Gr1, Ly6C, F4/80; conjugated to FITC, PE, PE/Cy5, APC, APC/Cy7, PE/Cy7, or Pacific Blue. Antibodies were purchased from Biolegend (San Diego, CA), Ebioscience (San Diego, CA), Abcam (Cambridge, MA), or R&D Systems (Minneapolis, MN). Samples were blocked for 10 min with 2.4G2 supernatant, stained for 20 min on ice, and washed with FACS buffer. Staining with Pacific Blue-conjugated Annexin V and 7AAD was performed as described by the manufacturer (Biolegend). After staining, peripheral blood samples were lysed of RBCs using FACS Lysing Buffer (BD Biosciences, San Jose, CA). Data were acquired using a FACStarPLUS or FACSCanto II flow cytometer (BD Biosciences) and analyzed using FlowJo software (Tree Star, Inc., Ashland, OR).

### Mixed bone marrow chimeras

c-FLIP^f/+^ or c-FLIP^+/+^ mice were lethally irradiated 4 hours prior to BM reconstitution via i.v. injection. Each mouse received either CD45.2^+^ c-FLIP^f/f^ Lysm-Cre BM or a 1∶1 mixture of CD45.2^+^ c-FLIP^f/f^ Lysm-Cre BM and CD45.2^+^/CD45.1^+^ c-FLIP^+/+^ BM. Thioglycollate-elicited PEC were examined 12 weeks post-transfer.

### Statistical analysis

All statistical analyses were performed using GraphPad Prism software (GraphPad, San Diego, CA). The results of *in vitro* experiments were analyzed using Student's *t*-tests and are presented as means. The results of *in vivo* experiments were analyzed using the Mann-Whitney test and are presented as geometric means. *, *p*<.05; **, *p*<.01; ***, *p*<.001. Error bars represent standard deviations (*in vitro* experiments) or 95% confidence intervals (*in vivo* experiments).

### Ethics Statement

This study was approved by the Duke University Institutional Animal Care and Use Committee (Protocol # A140-12-05). Euthanasia was performed by carbon dioxide inhalation followed by cervical dislocation.
